# Coil Embolization for Coronary Artery Perforation: A Retrospective Analysis of 110 Patients

**DOI:** 10.1155/2021/9022326

**Published:** 2021-11-12

**Authors:** Daisuke Hachinohe, Yoshifumi Kashima, Yuito Okada, Daitaro Kanno, Ken Kobayashi, Umihiko Kaneko, Takuro Sugie, Yutaka Tadano, Tomohiko Watanabe, Hidemasa Shitan, Takuya Haraguchi, Yusuke Morita, Nobuki Matsuna, Ryo Horita, Masanaga Tsujimoto, Tsuyoshi Takeuchi, Katsuhiko Sato, Tsutomu Fujita

**Affiliations:** ^1^Department of Cardiology, Asia Medical Group, Sapporo Heart Center, Sapporo Cardio Vascular Clinic, Sapporo, Japan; ^2^Cancer Epidemiology Program, University of Hawaii Cancer Center, Honolulu, HI, USA

## Abstract

**Objective:**

Coil embolization (CE) for coronary artery perforation (CAP) has not been thoroughly evaluated. This study aimed to evaluate the extent of myocardial damage and impact on cardiac function after CE for CAP.

**Methods:**

A total of 110 consecutive patients treated with CE for CAP were retrospectively identified. The degree of myocardial damage and impact on cardiac function were evaluated.

**Results:**

Forty-nine (44.5%) cases involved chronic total occlusions. A guidewire was the cause of perforation in 97 (88.2%) patients. The success rate of CE was 98.2%. Almost all patients were prescribed either antiplatelet drugs or anticoagulant medication or both. Patients with perforation types III and IV were found to be prone to creatinine kinase (CK) elevation and epicardial main vessel perforation, thereby causing myocardial damage. No changes were noted in the ejection fraction (EF) in patients with type V distal perforation and collateral channel perforation, while patients with perforation of the epicardial main vessel may show impaired cardiac function afterward.

**Conclusions:**

CE is safe and effective for treating CAP, especially when collateral channels and distal vessels are involved. Meanwhile, efforts should be taken to prevent CAP in epicardial main vessels since it may be difficult to treat with CS and cause myocardial damage when bailed out with CE leading to vessel sacrifice. We found that it was not necessary to change the anticoagulant regimen after CE owing to its ability to achieve robust hemostasis.

## 1. Introduction

Iatrogenic coronary artery perforation (CAP) is a rare but potentially lethal complication of percutaneous coronary intervention (PCI) [1] and may lead to tamponade, emergent pericardiocentesis, or surgical repair [2]. Once tamponade occurs, the in-hospital mortality grows to more than 5% even if pericardiocentesis is performed. Consequently, prevention of tamponade is essential to improve the acute survival rates after the detection of CAP [3]. In most cases, CAP can be managed with percutaneous procedures, including prolonged balloon sealing [4], suction through a microcatheter, blood clot embolization, fat tissue embolization, gel foam embolization [5], coil embolization (CE) [6], and covered stent (CS) implantation [3]. Determining the ideal method for the management of CAP depends on the perforation type [1], site, vessel size, and mechanism of the perforation. CS is a good therapeutic alternative to avoid surgical conversion, especially in cases of perforations involving large vessels and type III blow-out perforations, and can be life saving [3]. However, in cases of perforations of small vessels or those involving a significant side-branch vessel, or in distal perforations, there may be no use for CSs. In such cases, CE may be feasible and effective [6].

No data are available on the incidence of myocardial damage and effects on cardiac function after CE for CAP. This subject has important implications on the decision making regarding the treatment of CAP. Therefore, this study assessed the feasibility and safety of CE for CAP. To address this issue, we evaluate the incidence of myocardial damage and clinical outcomes and estimate the results of CE based on the perforation type, site, vessel size, and the cause of CAP. Furthermore, we examined the safety of CE defined as both the success rate of CE and the prescription of anticoagulant therapy after the procedure.

## 2. Materials and Methods

**Figure 1:**
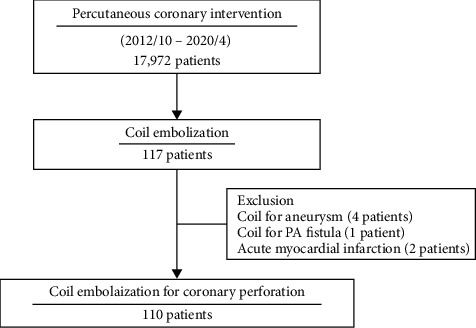
Study population.

Between October 2012 and April 2020, 17,972 patients underwent PCI in Sapporo Cardio Vascular Clinic, Sapporo, Japan. Of them, 117 patients were deployed coronary coils. Of the 117 recipients of coronary coils, 4, 1, and 2 patients who were deployed coils for coronary aneurysm, coronary-pulmonary artery fistula, and acute myocardial infarction (MI), respectively, were excluded from this study. Eventually, 110 patients were included in this study (Figure 1).

PCI was performed according to standard techniques, and the choice of technique was left to the operator's discretion. At the time of perforation, an echocardiogram was immediately performed to monitor pericardial effusion, and pericardiocentesis was performed in case of tamponade. Prompt balloon sealing and subsequent CS implantation were performed to prevent bleeding into the pericardial space in cases of type III perforations. In cases that involved significant bifurcation that needed to be protected or when the vessel size was small to not allow the use of CS, alternative options should be considered. For distal or collateral perforations, suction through a microcatheter was sometimes applied. If it failed to stop the bleeding, CE through a microcatheter was performed. A microcatheter was placed from the immediate proximal to the perforation site, and a microcoil was advanced through it using a guidewire. Three types of microcoils were used in this cohort. C-stopper (Solution, Yokohama, Japan) is a 0.014-inch microcoil that permits tight packing and immediate cross-sectional vessel occlusion owing to its flexibility and wave shape [7]. Hilal Embolization Microcoil (Cook, Bjaeverskov, Denmark) is a 0.018-inch coil with synthetic fibers that maximize thrombogenicity and can be delivered through a Finecross microcatheter [6]. TRUFILL (DePuy Synthes, Massachusetts, USA) is made from platinum alloy and synthetic fibers compatible with a 0.018-inch microcatheter. It is originally designed for neuroradiology and is longer than other microcoils used for CAP. After coiling, repeat coronary angiography after 10 min demonstrated an occlusion of the extravasation. The type and number of coils were as per the operator's discretion. Angiography was repeated to confirm the cessation of extravasation.

The baseline and procedural characteristics and clinical outcomes were retrospectively identified using our institutional database. The follow-up data were obtained using the medical records from our institution and those from other hospitals. All patients provided written informed consent for the procedure, subsequent follow-up, and analyses. This study was approved by our Institutional Review Board.

The co-primary endpoints were creatinine kinase (CK) levels on the day after the procedure, changes in the left ventricular ejection fraction (EF) during follow-up, pericardiocentesis to alleviate tamponade, emergent surgical repair, and in-hospital death. The lesion characteristics, including ostial stenosis, bifurcation stenosis, chronic total occlusion, and the American College of Cardiology/American Heart Association (ACC/AHA) classification, were defined as previously described [8]. CAP was defined using the Ellis criteria and its modified classification [1]. According to the European Society of Cardiology's third universal definition, periprocedural MI was defined as a rise in cardiac markers five times the upper limit of normal [9]. In this study, the epicardial vessel is defined as the nonseptal and nondistal vessel. The vessel diameter was measured using a quantitative angiographic analysis system CAAS software ver. 7.2 (Pie Medical Imaging, Maastricht, The Netherlands), as previously described [10].

Continuous variables are presented as mean ± standard deviation or medians and interquartile ranges, while categorical variables are expressed as numbers and percentages. All analyses were performed using IBM SPSS Statistics version 26.0 (IBM Corp. Armonk, NY, USA). Descriptive charts were created using R version 4.0.1 software and the ggplot2 package.

## 3. Results

The baseline clinical characteristics of the patients are shown in Table 1. The mean age of the study population was 75.9 ± 9.7 years. There was a higher number of male patients (69, 72.7%), and several patients had a history of coronary artery disease (43, 39.1% old MI; 69, 62.7% prior PCI; and 14, 12.7% prior coronary artery bypass grafting). Almost all patients underwent PCI for stable angina, while 4 (3.6%) were treated following unstable angina. Renal impairment (estimated glomerular filtration rate <60 mL/min/1.73 m^2^) was present in 61 (55.5%) patients.

Table 2 provides the angiographic and procedural characteristics. The target lesions were classified as ACC/AHA type B2 or C in 107 (97.3%) cases, and 49 (44.5%) cases involved chronic total occlusions (CTOs). More than half of the patients were deployed coils in the right coronary artery (45 patients, 40.9%), followed by the left circumflex artery (30, 27.3%), and the left anterior descending artery (29, 26.4%). Forty-three lesions (39.1%) were located at the distal site of the main vessel and 23 (20.9%), 11 (10.0%), and 5 (4.5%) at the distal portion of the branch vessel, mid of the main vessel, and mid of the branch vessel, respectively, whereas septal and nonseptal collaterals were involved in 8 (7.3%) and 20 (18.2%) cases, respectively, during CTO procedures. The reference vessel diameter measured by quantitative coronary angiography was 0.91 ± 0.34 mm. Type V perforations were seen in more than half the patients in this study. The percentage of the perforation type was different among perforation sites. Ellis type III perforations were found more in the mid-coronary perforation group (30.8%) than those found in the other groups. A guidewire was the cause of perforation in 97 (88.2%) patients, followed by rotational atherectomy in 7 (6.4%) cases, a microcatheter in 4 cases, and balloon dilatation in 1 case. C-stopper, Hilal Embolization Microcoil, and TRUFILL were used in 36 (32.7%), 66 (60.0%), and 8 (7.3%) cases, respectively, which were duplicately used in some cases. The average number of coils used was 4.52 ± 4.13. Protamine sulfate was administered in approximately half (61, 55.5%) of the patients. Cardiac tamponade, defined as the presence of pericardial fluid with hypotension and tachycardia, occurred in 7 (6.4%) cases who subsequently underwent pericardiocentesis. The success rate of CE was 98.2%. CSs were required in 3 cases, of whom 1 patient required extracorporeal membrane oxygenation and died on the same day from multiple organ failure. Another patient died of infection 1 month after PCI (Table 3). Intra-aortic balloon pumping became imperative in 1 patient. Postprocedural MI occurred in 6 (5.5%) patients. As for anticoagulant medication after perforation, dual antiplatelet therapy (APT) was prescribed in 95 (86.4%) patients and triple APT was prescribed in 1 patient (0.1%), while 12 patients were administered APT and oral anticoagulants, 1 patient was administered only direct oral anticoagulant, and another patient died before receiving either.

**Figure 2:**
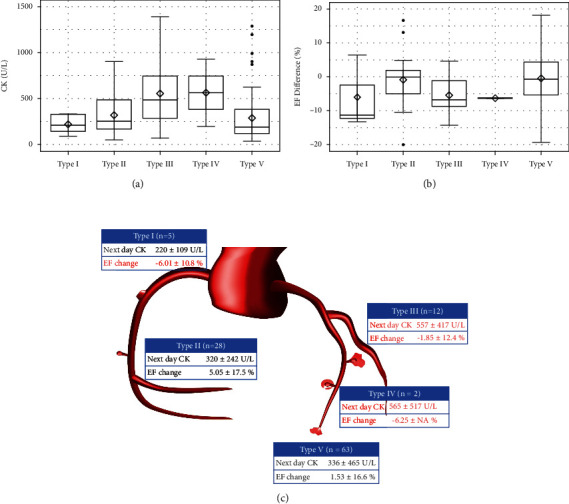
Box and whisker plot of creatinine kinase (CK) on the day after coil embolization (a), change in the ejection fraction (EF) from the baseline to follow-up (b), and schema (c) according to perforation type.

**Figure 3:**
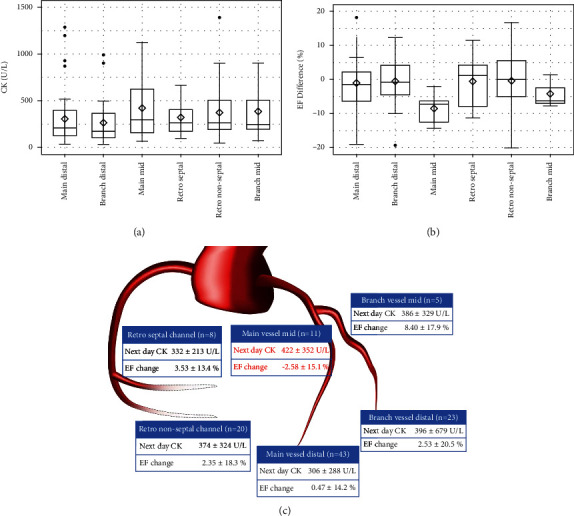
Box and whisker plot of creatinine kinase (CK) on the day after coil embolization (a), change in the ejection fraction (EF) from the baseline to follow-up (b), and schema (c) according to perforation site.

**Figure 4:**
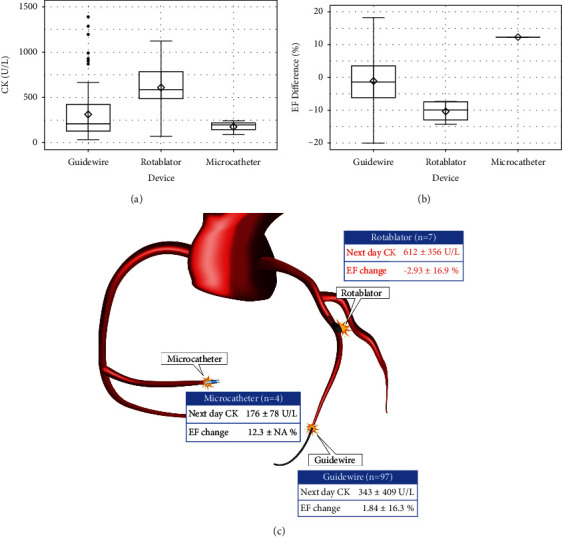
Box and whisker plot of creatinine kinase (CK) on the day after coil embolization (a), change in the ejection fraction (EF) from the baseline to follow-up (b), and schema (c) according to the cause of perforation.

**Figure 5:**
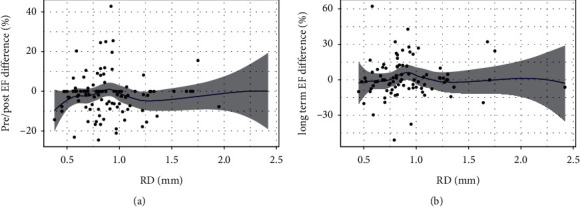
(a) Scatter plot of the reference diameter (RD) and creatinine kinase (CK); (b) scatter plot of the RD and ejection fraction (EF) change from the baseline to follow-up.

**Figure 6:**
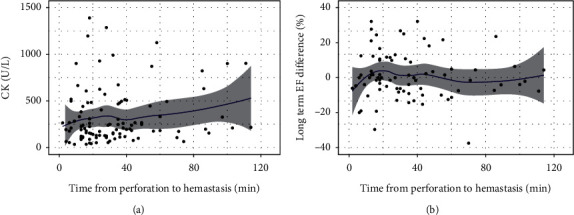
(a) Scatter plot of time from perforation to hemostasis and creatinine kinase (CK); (b) scatter plot of time from perforation to hemostasis and ejection fraction (EF) change from the baseline to follow-up.

Figure 2 shows the CK levels on the day after PCI, change in the EF from the baseline to follow-up (B), and schema (C) according to the perforation type. Patients with perforation types III and IV were prone to CK elevation (Figure 2(a)). No change was noted in the EF in patients with type V perforation, while in those with types I, III, and VI, it tended to decrease (Figure 2(b)). Mid perforation of the main vessel tended to cause myocardial damage (Figure 3). Interestingly, no change in the EF was seen in patients with collateral channel perforation. With respect to the cause of the perforation, wire perforation did not cause cardiac dysfunction, whereas perforation caused by Rotablator use was associated with CK elevation (Figure 4). As seen in Figure 5, cardiac function and EF were not affected by the perforated vessel size.  Figure 6 shows the scatter plots of time from perforation to hemostasis. A weak correlation was observed between the hemostasis time and CK elevation (Figure 6(a)), and the hemostasis time was poorly correlated with future cardiac function (Figure 6(b)).

## 4. Discussion

The main findings of the current study were as follows: (1) CE is safe and effective for treating CAP; (2) after CE, anticoagulant medications can be prescribed in the same way as after usual PCI; (3) perforation of the collateral channels and distal perforation do not cause CK elevation; and (4) an epicardial middle of main vessel perforation may aggravate cardiac damage and function, while the vessel size and hemostasis time do not affect the future cardiac function.

In this study, CE for CAP could successfully arrest bleeding in 98.2% cases and is therefore believed to be effective. Suction through a microcatheter, blood clot embolization [11], fat tissue embolization [12], and gel foam embolization [5] could be used instead of CE. However, the coil is visible under fluoroscopy, thereby allowing rapid and accurate delivery. Moreover, the coil has synthetic fibers that facilitate thrombogenicity and can secure better hemostasis than suction maneuver, blood clot [11], subcutaneous tissue [12], or gel foam embolization [5]. A concern after CAP is whether the administration of anticoagulants should be delayed or avoided due to the fear of recurrent bleeding. In this study, no recurrent bleeding was noted owing to the robust hemostasis achieved during the procedure. As proof of this, 95% of the patients received dual antiplatelet therapy and the others received anticoagulant medication with or without aspirin. This guaranteed hemostasis achieved using a coil for CAP may lower the incidence of thrombotic events after PCI; therefore, it eliminates the necessity of changing the anticoagulant regimen.

Regarding perforation during CTO PCI, Qin et al. reported that CE is feasible and effective for treating collateral perforation [6]. Theoretically, septal channel perforation is safer than nonseptal channel perforation, which may lead to tamponade [13]. Conversely, septal channel perforation is also associated with a small risk of septal hematoma leading to cardiogenic shock called “dry tamponade” [14]. In this study, we found that collateral channel perforation, including both septal and nonseptal channels, did not aggravate myocardial damage or cardiac function. Therefore, one should be fearless in treating collateral perforations during CTO PCI; overcoming this fear may improve the success rate of CTO PCI.

An important question that arises is whether large vessel perforation induces a more severe myocardial damage. Our data showed that the vessel diameter is not associated with CK elevation or a reduced EF. However, the type of perforation may be associated with myocardial damage. Al-Lamee et al. described the management of 56 patients with Ellis type III coronary perforation in two high-volume centers over 16 years, and the in-hospital mortality rate was 14.8% [15]. In this study, type III perforation occurred in 12 patients. Of them, pericardiocentesis was performed in 3 patients. In this cohort, 1 patient who died on the same day of the procedure had a type III perforation at the mid-portion of the left anterior descending artery involving a big diagonal branch. Extracorporeal membrane oxygenation was established with cardiopulmonary resuscitation and subsequently CSs were implanted; however, CS could not effectively achieve hemostasis. Perforation in 7 patients was caused by Rotablator use; however, the vessel was too small to deploy the CS. Three patients had small vessel perforation, and collateral perforation occurred in 2 patients. In routine practice, we frequently use CS to stop bleeding in cases of type III perforations. However, cases that involve significant bifurcation that needs to be protected or when the vessel size is small occasionally do not allow the use of CS. In such cases, CE may be the only alternative. CE could be used to treat type III perforations; however, due to the vast perfusion area, the myocardial damage is usually severe.

Regarding the perforation site, distal perforations do not cause myocardial damage. This result is consistent with the findings of previous reports [6]. Therefore, we should not hesitate to deploy coils in cases of type V perforations. Conversely, in epicardial vessel perforation, especially at the mid-portion of the main vessels, coil occlusion may cause MI, leading to a decreased EF during follow-up. In such lesions, balloon inflation or CS implantation to seal the perforated site should be considered first; however, if these fail or are found unfeasible, the use of a coil should be considered as a life-saving measure and avoid surgical intervention.

The hemostasis time may be associated with myocardial damage immediately after PCI; however, it may have a low impact on future cardiac function. Myocardial damage may be associated with the mechanism of perforation, perforation site, and perfusion area. If type III perforation occurs, especially in the epicardial vessel and mid-portion of the vessels, physicians should take urgent steps to arrest the bleeding as soon as possible and closely monitor the pericardial effusion. In cases of cardiac tamponade, emergency pericardiocentesis must be performed. By contrast, if the perforation is not of the blow-out type, one needs not proceed hastily with the PCI if the vital signs are stable, and tamponade is not predicted.

The strength of this study was the availability of more than 100 samples for CE and with robust outcomes, such as the combination of CK and EF to evaluate cardiovascular function. However, this study had some limitations. First, this study was based on observational data from a high-volume center in Japan. The results were most likely to be influenced by the nonrandomized assignment and the presence of some confounding factors. We compared the clinical outcomes based on the perforation type, site, vessel size, and the cause of CAP. However, the population size was underpowered to reach statistical conclusions. Second, CK was the only biomarker measured on the day after the procedure, whereas CK-MB and troponins were not available. The incidence of periprocedural MI using only CK will be much less sensitive than that of other biomarkers. Third, we demonstrated that CE is safe and effective in patients with CAP, especially for perforations involving collateral channels. Further studies are needed to establish the best treatment for CAP. Owing to the difficulties in conducting randomized controlled trials on patients treated with CE or those with CAP due to the paucity of patients, larger-scale multicenter registries are warranted.

## 5. Conclusion

CE is safe and effective for treating CAP, especially when collateral channels and distal vessels are involved. Meanwhile, efforts should be taken to prevent CAP in epicardial main vessels since it may be difficult to treat using CS with vessel sacrifice to block extravasation. It is unnecessary to change the anticoagulant regimen after CE owing to its ability to achieve robust hemostasis.

## Figures and Tables

**Table 1:** Baseline clinical characteristics and medication.

	110 patients
*Clinical*	
Age (years)	75.9 ± 9.7
Male sex, *n* (%)	69 (72.7%)
Body mass index (kg/m^2^)	23.2 ± 3.3
Hypertension, *n* (%)	77 (70.0%)
Hyperlipidemia, *n* (%)	75 (68.2%)
Diabetes mellitus, *n* (%)	40 (36.4%)
Smoker, *n* (%)	56 (50.9%)
Hemodialysis, *n* (%)	13 (11.8%)
Old myocardial infarction, *n* (%)	43 (39.1%)
Prior history of PCI, *n* (%)	69 (62.7%)
Prior history of CABG, *n* (%)	14 (12.7%)
Atrial fibrillation, *n* (%)	27 (24.5%)
Peripheral artery disease, *n* (%)	11 (10.0%)
Stroke, *n* (%)	10 (9.1%)
COPD, *n* (%)	4 (3.6%)

*Clinical diagnosis*	
Stable angina, *n* (%)	106 (96.4%)
Unstable angina, *n* (%)	4 (3.6%)

*Laboratory data*	
LDL cholesterol (mg/dl)	99.6 ± 35.7
HbA1c (%)	6.1 ± 0.8
eGFR (mL/min/1.73 m^2^)	52.8 ± 24.9
eGFR <30 mL/min/1.73 m^2^, *n* (%)	21 (19.1%)
eGFR <60 mL/min/1.73 m^2^, *n* (%)	61 (55.5%)
Left ventricular ejection fraction (%)	58.5 ± 12.8

Values are expressed as numbers (%) or means (±SD). PCI, percutaneous coronary intervention; CABG, coronary artery bypass graft; COPD, chronic obstructive pulmonary disease; LDL, low-density lipoprotein; eGFR, estimated glomerular filtration rate.

**Table 2:** Coronary angiographic and procedural characteristics.

	110 patients
Left main complex, *n* (%)	3 (2.7%)
Left anterior descending, *n* (%)	29 (26.4%)
Left circumflex, *n* (%)	30 (27.3%)
Right coronary artery, *n* (%)	45 (40.9%)
Other vessels, *n* (%)	5 (4.5%)
In-stent restenosis, *n* (%)	9 (8.2%)
Type B2/C lesion, *n* (%)	107 (97.3%)
Eccentric lesion, *n* (%)	85 (77.3%)
Diffuse lesion, *n* (%)	96 (87.3%)
Lesion angulation ≧ moderate, *n* (%)	17 (15.5%)
Proximal tortuosity ≧ excessive, *n* (%)	67 (8.7%)
Calcified lesion, *n* (%)	54 (49.1%)
Thrombus laden, *n* (%)	2 (1.8%)
Bifurcation, *n* (%)	41 (37.3%)
Ostial lesion, *n* (%)	6 (5.5%)
Chronic total occlusion, *n* (%)	49 (44.5%)

*Sheath size*	
6-french, *n* (%)	52 (47.3%)
7-french, *n* (%)	24 (21.8%)
8-french, *n* (%)	34 (30.9%)

*Approach site*	
Femoral, *n* (%)	71 (64.5%)
Radial, *n* (%)	38 (34.5%)
Brachial, *n* (%)	1 (0.9%)
Imaging device, *n* (%)	24 (21.8%
IVUS, *n* (%)	104 (94.5%)
OCT, *n* (%)	1 (0.9%)
Debulking device, *n* (%)	4 (0.5%)
Directional coronary atherectomy, *n* (%)	0 (0%)
Rotational atherectomy, *n* (%)	24 (21.8%)
Excimer laser coronary atherectomy, *n* (%)	0 (0%)

*Perforation classification*	
Type I, *n* (%)	5 (4.5%)
Type II, *n* (%)	28 (25.5%)
Type III, *n* (%)	12 (10.9%)
Type IV, *n* (%)	2 (1.8%)
Type V, *n* (%)	63 (57.3%)

*Perforation site*	
Main vessel distal, *n* (%)	43 (39.1%)
Main vessel middle, *n* (%)	11 (10.0%)
Branch vessel distal, *n* (%)	23 (20.9%)
Branch vessel middle, *n* (%)	5 (4.5%)
Septal collateral channel, *n* (%)	8 (7.3%)
Nonseptal collateral channel, *n* (%)	20 (18.2%)

*Cause of perforation*	
Guidewire, *n* (%)	97 (88.2%)
Balloon, *n* (%)	1 (0.9%)
IVUS, *n* (%)	0 (0%)
Stent, *n* (%)	0 (0%)
Microcatheter, *n* (%)	4 (3.6%)
Directional coronary atherectomy, *n* (%)	0 (0%)
Rotational atherectomy, *n* (%)	7 (6.4%)
Excimer laser coronary atherectomy, *n* (%)	0 (0%)
Reference diameter of perforated vessel (mm)	0.91 ± 0.34
Covered stent, *n* (%)	3 (2.7%)
Protamine sulfate, *n* (%)	61 (55.5%)
Cardiac tamponade, *n* (%)	7 (6.4%)
Pericardiocentesis, *n* (%)	7 (6.4%)
Surgical repair, *n* (%)	0 (0%)
Intra-aortic balloon pumping, *n* (%)	1 (0.9%)
Extracorporeal membrane oxygenation, *n* (%)	1 (0.9%)
Impella, *n* (%)	0 (0%)
The number of microcoils, *n*	4.52 ± 4.13
Coiling success, *n* (%)	108 (98.2%)

Values are expressed as numbers (%) or means (±SD). Type B2/C, according to the American College of Cardiology/American Heart Association classification system; IVUS, intravascular ultrasound; OCT, optical coherent tomography.

**Table 3:** Medication and outcomes after percutaneous coronary intervention.

	110 patients
*Medication*	
Aspirin, *n* (%)	108 (99.1%)
Ticlopidine, *n* (%)	4 (3.7%)
Clopidogrel, *n* (%)	44 (40.4%)
Prasugrel, *n* (%)	55 (50.5%)
Ticagrelor, *n* (%)	0 (0%)
Cilostazol, *n* (%)	5 (4.6%)
Warfarin, *n* (%)	10 (9.2%)
DOAC, *n* (%)	13 (11.8%)
Single APT	0 (0%)
Single APT + OAC	3 (2.7%)
Dual APT	95 (86.4%)
Dual APT + OAC	9 (8.2%)
Triple APT	1 (0.1%)
Only DOAC	1 (0.1%)

*Clinical outcomes*	
In-hospital death, *n* (%)	2 (1.8%)
Creatine kinase of the next day (U/L)	352 ± 404
Postprocedural MI, *n* (%)	6 (5.5%)
Left ventricular EF of the next day (%)	57.1 ± 12.1
EF changes from baseline to follow-up (%)	0.69 ± 8.05

Values are expressed as numbers (%) or means (±SD). APT, antiplatelet therapy; OAC, oral anticoagulants; DOAC, direct oral anticoagulants; MI, myocardial infarction; EF, ejection fraction.

## Data Availability

The data used to support this study are available on request from the corresponding author.
